# Clinical Characteristics of Patients with Pyogenic Vertebral Osteomyelitis and Concurrent Infections and Their Clinical Outcomes

**DOI:** 10.3390/jpm12040541

**Published:** 2022-03-29

**Authors:** Donghyun Kim, Jihye Kim, Taehwan Kim

**Affiliations:** 1 Spine Center, Department of Orthopedics, Hallym University Sacred Heart Hospital, Hallym University College of Medicine, Anyang 14068, Korea; dong8@hallym.or.kr; 2 Division of Infection, Department of Pediatrics, Kangdong Sacred Heart Hospital, Hallym University College of Medicine, Seoul 05355, Korea; jihyewiz17@kdh.or.kr

**Keywords:** concurrent infection, spondylodiscitis, pyogenic vertebral osteomyelitis, recurrence, mortality, multiple

## Abstract

Patients with pyogenic vertebral osteomyelitis (PVO) often develop concurrent infections, and a significant number of these patients show rapid deterioration in their medical condition, leading to mortality without PVO-related structural instability or neurological deficits. To improve clinical outcomes, we investigated the clinical presentation and treatment outcomes of patients with PVO and concurrent infections. This study included 695 patients with PVO, of which 175 (25%) had concurrent infections and 520 (75%) did not. The clinical characteristics of the two groups were compared, and multivariable analysis was performed to identify the association between concurrent infections and clinical outcomes. Patients with concurrent infections were older and had more comorbidities than those without. Moreover, there were significant intergroup differences in the anatomical involvement of PVO, and patients with concurrent infections had a higher number of regions involved more frequently than those without concurrent infections (15% vs. 6%). In contrast, patients with concurrent infections showed a lower degree of focal invasiveness, including a lower incidence of posterior abscess (47% vs. 59%; *p* = 0.008) and fewer neurological impairments according to the American Spinal Injury Association grade (*p* < 0.001) than those without concurrent infections. The causative organisms also differed significantly between the two groups, and patients with concurrent infections had a greater proportion of Gram-negative infections (31% vs. 16%, respectively) and a smaller proportion of methicillin-resistant *S. aureus* infections than those without concurrent infections (6% vs. 24%). Consequently, their clinical outcomes were significantly different, and patients with concurrent infections showed lower recurrence and higher mortality rates. We investigated the 1-year recurrence and mortality rates and their 95% confidence intervals according to the types of concurrent infections and their time of diagnosis and found variations in these parameters. Our results, based on a large number of patients, can be practically used as a reasonable reference to warn clinicians of the clinical risks of concurrent infections in patients with PVO and to help predict their clinical outcomes.

## 1. Introduction

Most patients with pyogenic vertebral osteomyelitis (PVO) without structural instabilities or neurological deficits have an uneventful clinical course [1], and mortality is not significantly influenced by PVO if appropriate antibiotics are administered for sufficient periods. However, the diagnosis of PVO is frequently delayed in elderly patients with comorbidities [2], and they often present with concurrent infections in other organs. In our clinical practice, a significant number of patients with PVO and concurrent infections show rapid deterioration of their condition, leading to mortality even in the absence of structural instability or neurological deficits resulting from PVO. A retrospective study of patients with PVO and liver cirrhosis showed that concurrent infections were present in 56.5% of patients, and concurrent infection was an independent predictor of mortality [3].

Concurrent infections after the diagnosis of PVO are expected to be rare owing to the use of intravenous antibiotics, except for *Clostridium difficile* or fungal infections, which can paradoxically occur with the long-term use of antibiotics. However, in clinical practice, concurrent infections are frequently encountered not only before the diagnosis of PVO but also after administering antibiotics for PVO. Theoretically, concurrent infections can occur in other organs even after the use of antibiotics for PVO because of the inadequate use of antibiotics, as in patients with culture-negative PVO, or when a new causative organism is incidentally introduced to other organs, such as in patients with concurrent urinary tract infections (UTI) or aspiration pneumonia [4].

Patients with PVO and concurrent infections are expected to have adverse clinical outcomes for the following reasons. First, concurrent infections usually occur in elderly patients with comorbidities who are already at a higher risk of adverse clinical outcomes. [5] Second, owing to pre-existing severe pain and reduced mobility in patients with PVO, newly developed symptoms and signs resulting from concurrent infections are often neglected. Therefore, concurrent infections are frequently detected in advanced stages. Third, owing to the avascularity of the intervertebral space, attaining an adequate concentration of antibiotics within the infected vertebral structures may take a long time. The resultant persistent spinal infection and bacteremia negatively influence the clinical outcomes of other vital organs with concurrent infections.

To improve the clinical outcomes of patients with PVO and concurrent infections, their precise clinical presentation and treatment outcomes must first be evaluated. However, to date, no studies have investigated these factors. To the contrary, previous studies investigating the clinical outcomes of patients with PVO regarded concurrent infections as a confounder that affected the results and they excluded patients with concurrent infections to reduce bias. [4,6,7] From this respect, our retrospective study aimed to investigate the epidemiology of concurrent infections in patients with PVO and compare their clinical presentation and treatment outcomes with those of patients without concurrent infections. In our retrospective study, a causal relationship between clinical outcome and concurrent infection could not be established; instead, we focused on presenting precise clinical presentations and outcomes of patients with PVO and concurrent infection.

## 2. Patients and Methods

### 2.1. Study Design and Ethics

**Figure 1:**
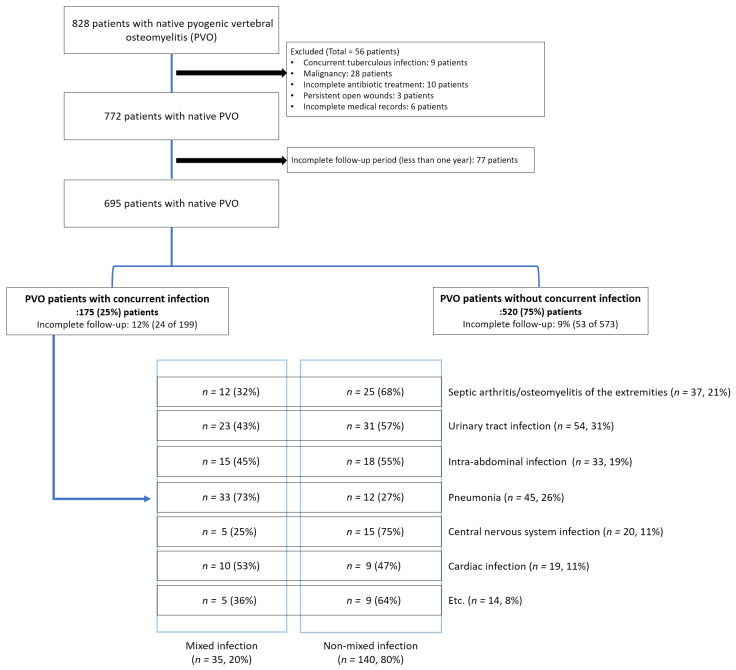
Patient enrollment.

This retrospective comparative study included patients who visited our institution between January 2013 and January 2021. Patients diagnosed with and treated for PVO were initially included in the study. PVO was diagnosed based on the findings of previous studies [3,7,8]; accordingly, only culture-positive cases were included. Patients with a history of spinal instrumentation at the site of infection were excluded. Additionally, we excluded patients who received incomplete antibiotic treatment for PVO. PVO could have been incidentally diagnosed while treating concurrent infections; therefore, the appropriate duration of antibiotic therapy could not be precisely determined in such cases. Then, we excluded the following patients (except for those who had died within 6 weeks): those in whom the total duration of antibiotic use was less than 6 weeks and those in whom the duration of antibiotic use was less than 4 weeks after the incidental diagnosis of PVO. Additionally, we excluded the following patients: patients with concurrent tuberculous infections, malignancy, persistent open wounds, and pressure ulcers, and those with incomplete medical records or follow-up periods (less than 1 year) (Figure 1).

This study was designed and conducted in accordance with the Strengthening the Reporting of Observational Studies in Epidemiology (STROBE) guidelines. This study was approved by the Institutional Review Board of Hallym University Sacred Heart Hospital (2021-01-002-001), and the need for informed consent was waived by the Institutional Review Board of Hallym University. All procedures were performed in accordance with the relevant guidelines and regulations.

### 2.2. Definitions and Evaluation of Cconcurrent Infections in Patients with PVO

The presence of concurrent infections was defined according to previous studies [3].

(1) Cardiac infection: Infective endocarditis was diagnosed using the modified Duke criteria [9].
(2) Pneumonia: Pneumonia was diagnosed in patients with at least one respiratory symptom and one of the following signs: rales and/or crepitation on auscultation, at least one sign of infection in the absence of antibiotic therapy, presence of pulmonary infiltrate on radiological imaging, or positive sputum culture [3].
(3) Intra-abdominal infection: Based on a previous study [10], intra-abdominal infections, including single-organ infections, peritonitis, and abdominal abscesses were considered. Additionally, infectious enterocolitis was considered in patients with diarrhea; those showing leukocytes in stool samples; pathogen-positive stool cultures for *Salmonella*, *Shigella*, *Yersinia*, *Campylobacter*, and pathogenic *Escherichia coli*; and those showing a positive *Clostridium difficile* stool assay [3].
(4) UTI: This only included patients with symptomatic UTI characterized by bacteriuria (urinary pathogens with ≥10^5^ colony-forming units/mL) in the presence of genitourinary symptoms [11].
(5) Septic arthritis or osteomyelitis of the extremities: Septic arthritis was diagnosed if the synovial fluid leukocyte count was >50,000 cells/μL or in the presence of a positive synovial fluid culture [12], whereas osteomyelitis was diagnosed based on typical radiological and magnetic resonance imaging (MRI) findings or positive culture results [13,14,15].
(6) Central nervous system infection: This was diagnosed by cerebrospinal-fluid-confirmed bacterial meningomyelitis and abscess formation in the brain and spinal cord using MRI [16].

Radiological (chest and abdomen radiography) and laboratory examinations, including inflammatory markers and urinalysis, were routinely performed biweekly on fixed days for all patients with PVO (Monday and Thursday) at our institute. Based on these results, infectious disease specialists determined further evaluation and management plans for combined infections. Precise protocols for the medico-surgical treatment of PVO have been presented in our previous reports [5,6].

### 2.3. Covariates and Treatment Outcomes

Demographic data and medical comorbidities of patients with PVO were retrieved from medical records, and Charlson comorbidity index (CCI) scores were calculated accordingly [17]. Anatomical involvement of the infections was assessed by the primary region involved, location of abscess (epidural, anterior or posterior to the epidural space), and number of infected bodies, and graded using the system described by Pola et al. [3,7,8,18]. Neurological impairment was assessed using the American Spinal Injury Association (ASIA) impairment scale. Data regarding causative organisms, laboratory test results, medical treatment including the type and duration of antibiotics, and surgical treatment including early (within 6 weeks after diagnosis) spinal instrumentation were also retrieved.

Recurrence was defined as the presence of recurrent symptoms and signs after the completion of the initial antibiotic course and administration of a second course of intravenous antibiotics [3,7,8]. Mortality was assessed as 90-day and 1-year mortality rates.

### 2.4. Statistical Methods

The clinical characteristics of patients with PVO and concurrent infections were statistically evaluated using the following three steps.

First, we compared the clinical data, including baseline characteristics and precise infection and treatment profiles for patients with and without concurrent infections. Continuous variables were compared using the independent t-test, whereas categorical variables were compared using the χ^2^ test or the linear-by-linear association test. Kaplan–Meier survival curves were generated to compare the survival curves between the two groups, and the log-rank test was additionally used for comparison. Multivariable logistic regression analysis was used to identify the association between concurrent infection and clinical outcomes, including recurrence and 90-day and 1-year mortality, adjusting for variables that showed intergroup differences, with statistical significance set at *p* < 0.05.

Second, subgroup analyses were performed to identify the association between each type of concurrent infection and clinical outcomes using multivariable logistic regression analysis. In the subgroup analysis, multivariable adjustment was additionally performed for other types of concurrent infections that showed intergroup differences between patients with and without the target concurrent infections. Multicollinearity among each type of concurrent infection was estimated using the variance inflation factor (VIF).

Finally, we further divided patients with single concurrent infections into two groups according to the time of diagnosis of concurrent infections: those who had concurrent infections before and after the diagnosis of PVO. We calculated the 1-year recurrence and mortality rates and their 95% confidence intervals according to the types of concurrent infection and their timing of diagnosis using the Hosmer–Lemeshow method.

Statistical tests were two-tailed, and *p*-values < 0.05 indicated statistical significance. Statistical analyses were performed using SPSS version 25 (IBM Corp., Armonk, NY, USA).

## 3. Results

This study included 695 patients with PVO (Figure 1), of whom 368 (53%) were women, with a mean age of 69.2 years (range, 20–92 years). The patients were divided into two groups based on the presence (*n* = 175, 25%) or absence (*n* = 520, 75%) of concurrent infections (Figure 1).

### 3.1. Comparison of Baseline Characteristics

The most common type of concurrent infection was UTI (31%, 54/175), followed by pneumonia (26%, 45/175) (Figure 1). Musculoskeletal infections were identified in 21% (37/175) of the patients, whereas septic arthritis and osteomyelitis of the extremities were observed in 29 and 14 patients, respectively (six patients had both infections). Intra-abdominal infections were identified in 19% (33/175) of the patients, of whom 33% (11/33) had *Clostridium difficile* infections. Central nervous system infections were diagnosed in 11% (20/175) of patients, of which 8 and 14 patients were diagnosed with brain abscess and meningomyelitis (two patients had both infections), respectively. Cardiac infection was diagnosed in 11% (19/175) of patients. The remaining 14 patients were diagnosed with the following concurrent infections: cellulitis or myofascitis of the extremities (*n* = 8), facial bone infections, including dental infection (*n* = 3), and pelvic abscess (*n* = 3).

Patients with concurrent infections were significantly older (72.5 vs. 68.1 years; *p* < 0.001) and had higher CCI scores (3.0 vs. 2.1; *p* < 0.001) than those without concurrent infections (Table 1).

### 3.2. Comparison of Infection Profiles

In both groups, the lumbosacral region was the most common primary site of infection (Table 2). However, multiple spinal lesions were more frequently observed in patients with concurrent infections (15%) than in those without (6%) (Table 2). Despite the similar proportions of epidural abscesses (*p* = 0.830) and abscesses anterior to the epidural space (*p* = 0.648), abscesses posterior to the epidural space were more frequently observed in patients without concurrent infections than in those with concurrent infections (47% vs. 59%; *p* = 0.008). In addition, patients without concurrent infections showed more severe neurological impairment, as assessed by the ASIA grade (*p* < 0.001).

The most common causative organism was *Staphylococcus aureus* (49%, 344/695). However, methicillin-sensitive *S. aureus* infections were prevalent in patients with concurrent infections (38% vs. 27%), whereas methicillin-resistant *S. aureus* (MRSA) infections were more prevalent in those without concurrent infections (6% vs. 24%) (Table 2). The precise causative organisms of the cohort patients are presented in Supplementary Table S1. Intergroup differences were not observed in the severity of infection, number of infected vertebral bodies, or laboratory values of the three inflammatory markers (Table 1 and Table 2).

### 3.3. Comparison of Treatment Profiles

**Figure 2:**
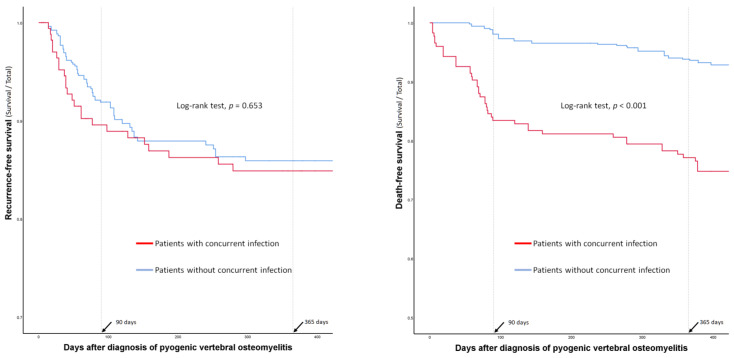
Comparison of recurrence- and death-free survival curves between the patients with and without concurrent infections.

Surgical treatment, including early spinal instrumentation (within 6 weeks of PVO diagnosis), was more frequently performed in patients without concurrent infections (Table 3). However, complications associated with surgery or the long-term use of antibiotics were not significantly different between the groups (Table 3). The duration of antibiotic administration and hospital stay were significantly longer in patients without concurrent infections than in those with concurrent infections. There were no significant differences in recurrence rates or recurrence-free survival between the two groups (14% vs. 14%; *p* = 0.965) (Table 3 and Figure 2). However, the 90-day mortality (17% vs. 2%; *p* < 0.001) and 1-year mortality (23% vs. 6%; *p* < 0.001) rates were significantly higher in patients with concurrent infections than in those without (Table 3 and Figure 2).

### 3.4. Association between Concurrent Infections and Clinical Outcomes in the Whole Cohort

Although concurrent infections were not associated with recurrence in univariate analysis, they were associated with a lower risk of recurrence in multivariable analysis (odds ratio (OR) = 0.49, *p* = 0.030) (Table 4). In contrast, concurrent infection was consistently associated with mortality, and patients with concurrent infection showed increased risks for 90-day (OR = 7.70, *p* < 0.001) and 1-year mortality (OR = 4.10, *p* < 0.001).

### 3.5. Association between Individual Concurrent Infections and Clinical Outcomes: Subgroup Analysis

In the subgroup analysis, the six types of single concurrent infections were not associated with recurrence (Table 4). However, cardiac infection was significantly associated with higher 90-day mortality (OR = 6.17, *p* = 0.017), and central nervous system infection was a consistent risk factor for 90-day (OR = 27.50, *p* < 0.001) and 1-year mortality (OR = 14.05, *p* < 0.001) (Table 4).

Subgroup analysis was also performed for patients with multiple concurrent infections (*n* = 35, 20%; Figure 1). Among the 35 patients with multiple concurrent infections, most (71%, 25/35) had two concurrent infections simultaneously. However, eight patients had three types of concurrent infections, and two patients had four types of concurrent infections. Multiple concurrent infections were consistently associated with recurrence (OR = 3.38, *p* = 0.011), 90-day mortality (OR = 9.42, *p* < 0.001), and 1-year mortality (OR = 3.22, *p* = 0.020) (Table 4). The VIF method was used to identify multicollinearity among the individual types of concurrent infections, and all VIF values were lower than 2.5 (Table 5).

**Figure 3:**
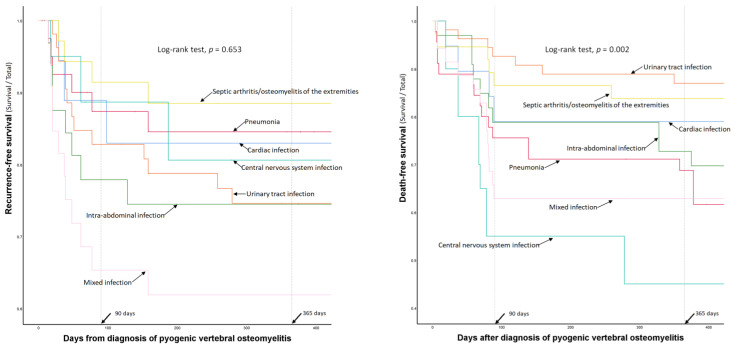
Comparison of recurrence- and death-free survival curves according to the types of concurrent infections.

The recurrence- and death-free survival curves of patients with single and multiple concurrent infections are presented in Figure 3.

### 3.6. One-Year Recurrence and Mortality Rates of Patients with Concurrent Infections: Subgroup Analysis According to the Timing of Diagnosis

**Figure 4:**
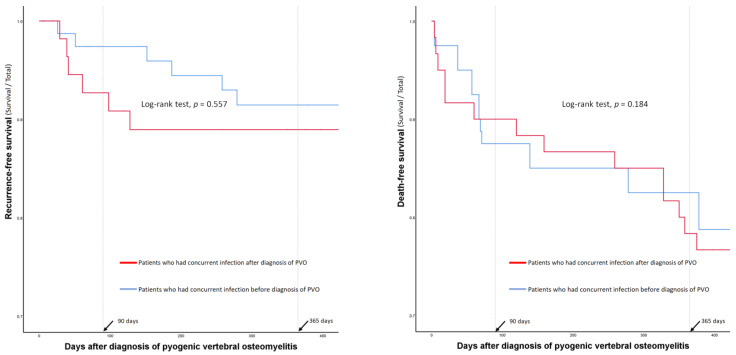
Comparison of recurrence- and death-free survival curves according to the diagnostic timings of the concurrent infections.

The log-rank test did not show significant differences in recurrence- and death-free survival according to the diagnostic timing in the entire cohort with concurrent infections (*n* = 175, Figure 4).

**Figure 5:**
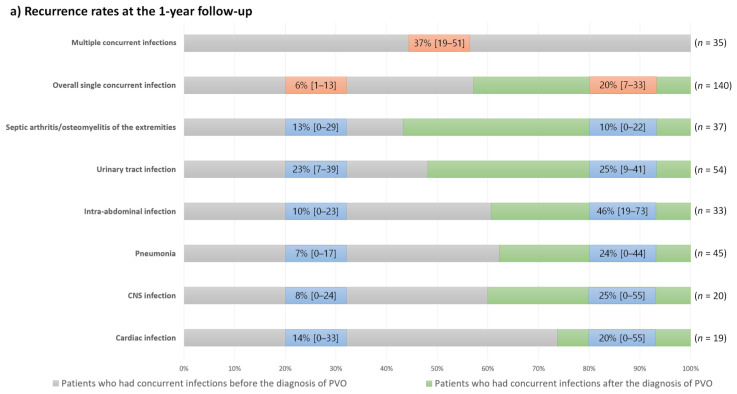

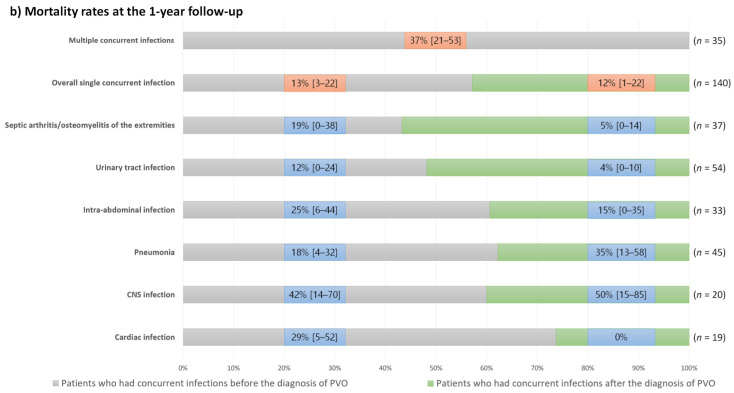
One-year recurrence and mortality rates and their 95% confidence intervals in the patients with concurrent infections according to the types of concurrent infections and their diagnostic timings; (**a**) Recurrence rates at the 1-year follow-up; (**b**) Mortality rates at the 1-year follow-up.

However, the 1-year recurrence and mortality rates of patients with individual types of concurrent infection showed varying degrees of difference according to their diagnostic timings (Table 5 and Figure 5).

Except for patients with septic arthritis/osteomyelitis of the extremities, those who had concurrent infections after the diagnosis of PVO had a higher recurrence rate than those who had concurrent infections before the diagnosis of PVO. Conversely, mortality rates were generally higher in patients with concurrent infections before the diagnosis of PVO. However, patients with central nervous system infections showed high mortality rates regardless of the diagnostic timing of concurrent infections, and mortality rates were higher in patients who had concurrent pneumonia after the diagnosis of PVO than in those with pneumonia before the diagnosis of PVO.

## 4. Discussion and Conclusions

Knowledge of the clinical characteristics of patients with PVO and their concurrent infections is a prerequisite for improving their clinical outcomes; hence, we investigated their clinical presentations and treatment outcomes. Of the 695 patients with PVO, 175 (25%) had concurrent infections, and they were older and had more comorbidities than those without concurrent infections. Significant intergroup differences were observed in the anatomical involvement of the PVO. Although the common primary region of infection in both groups was the lumbosacral region, patients with concurrent infections had multiple infected regions more frequently than those without concurrent infections (15% vs. 6%, Table 2). In contrast, patients with concurrent infections showed a lower degree of focal invasiveness, including a lower incidence of posterior abscess (47% vs. 59%; *p* = 0.008) and fewer neurological impairments according to the ASIA grade (*p* < 0.001) than those without concurrent infections (Table 2). As a result, surgical treatment, including early spinal instrumentation, was less frequently performed in patients with concurrent infection due to their lower degree of local invasiveness (Table 3).

The causative organisms also significantly differed between the two groups, and patients with concurrent infections had a higher proportion of Gram-negative infections (31% vs. 16%, respectively) and a lower proportion of MRSA infections compared to those without concurrent infections (6% vs. 24%, Table 2). Multivariable analysis identified that, in patients with PVO, the presence of concurrent infections was associated with lower recurrence of infection and higher mortality rates (Table 4). In contrast, multiple concurrent infections are associated with a higher risk of recurrence and mortality. Based on these significant associations between concurrent infections and clinical outcomes, we calculated the 1-year recurrence and mortality rates and their 95% confidence intervals according to single or multiple concurrent infections and their diagnostic timing (Figure 5).

The distinct differences in the anatomical involvement of infection and neurological impairment between patients with and without concurrent infections can be explained by differences in the types of causative organisms. In our study, Gram-negative bacteria were more frequently observed in patients with concurrent infections than in those without. Infections caused by Gram-negative bacteria are thought to be more inflammatory than those caused by Gram-positive bacteria [19], and the expected higher degree of systemic inflammation in patients with concurrent infections might paradoxically have enabled the detection of the infection before the bacteria significantly destroyed the vertebral structure and caused neurological impairment. Conversely, patients without concurrent infections, whose infections were predominantly caused by Gram-positive organisms, may have had a less eventful clinical course. Therefore, these infections may have a higher chance of being diagnosed after structural instability and neurological impairment develop. Owing to this increase in local invasiveness, the duration of antibiotic use was significantly longer in patients without concurrent infections (45 vs. 53 days; *p* < 0.001) (Table 2).

In accordance with these differences in clinical presentation, the treatment outcomes differed significantly. MRSA infections and undrained abscesses were closely associated with the recurrence of PVO [3,4,7,8,20], and patients with concurrent infections who presented with a lower proportion of MRSA infections and fewer abscesses (Table 2) showed a lower risk of recurrence (Table 4). In contrast, the mortality rate is higher in patients with concurrent infections. Considering their different clinical presentations, the reasons for the higher mortality in patients with concurrent infections could be as follows. First, the patients with concurrent infections were older and had more comorbidities, which could have negatively influenced their survival. Second, infections caused by Gram-negative bacteria are associated with poorer prognosis owing to a more severe inflammatory response and sepsis compared to those caused by Gram-positive bacteria [19], and the higher proportion of Gram-negative causative organisms in patients with concurrent infections could also have negatively influenced their survival.

The clinical outcomes of patients with concurrent infections varied considerably according to the type of infection (Figure 5). Even in patients with the same type of concurrent infection, recurrence and mortality rates differed according to the diagnostic timing of concurrent infection. Therefore, we presented the 1-year recurrence and mortality rates in our cohort according to these factors (Figure 5). Generally, patients who had concurrent infections after the diagnosis of PVO had a higher recurrence rate than those who had concurrent infections before the diagnosis of PVO. Conversely, mortality rates are generally higher in patients with concurrent infections before the diagnosis of PVO. Based on these findings, the higher 1-year mortality rates of patients with concurrent nervous system infections, pneumonia developing after the diagnosis of PVO (mostly identified as aspiration pneumonia), intra-abdominal and cardiac infections developing before the diagnosis of PVO, and multiple concurrent infections should be noted.

Our results should be interpreted carefully considering certain study limitations. This study aimed to investigate the clinical characteristics of patients with PVO and concurrent infection. Therefore, a causal relationship between concurrent infections and clinical outcomes was not established in our study. In addition to the various factors included, unknown confounders such as individuals’ bone quality or physical activity might have influenced the association between concurrent infections and clinical outcomes. Therefore, further high-quality studies that include additional clinical information are required to precisely understand the cause of higher mortality in patients with concurrent infections. Second, the minimal follow-up period of our study was 1 year, and 77 of the 695 (11%, Figure 1) patients were lost to follow-up. Recurrence or mortality associated with infection could have occurred in patients who were lost to follow-up or after 1 year. Therefore, a long-term, prospective study may have yielded different results. Third, we did not consider the concordance of causative organisms for the definition of concurrent infections because we did not perform separate biopsies for each causative organism of PVO and concurrent infections for all patients. Further studies that can identify the concordance of causative organisms in patients with PVO and concurrent infections might help understand the reasons behind their different clinical characteristics. Despite these limitations, the higher mortality rate of patients with concurrent infections should not be underestimated. In clinical practice, the worst-case estimate of recurrence and mortality rates and their 95% confidence intervals (Figure 5) should be considered to prevent adverse clinical outcomes.

In conclusion, our study identified that a quarter of the patients with PVO presented with concurrent infections. These patients were older and had more comorbidities than those without concurrent infection. There were also significant intergroup differences in the anatomical involvement of the infections, degrees of neurological impairment, and types of causative organisms. As a result, the clinical outcomes were significantly different between patients with and without concurrent infections. In addition, we investigated the 1-year recurrence and mortality rates, and their 95% confidence intervals according to the types of concurrent infections and their diagnostic timings, and recurrence and mortality rates varied according to them. Although a causal relationship between concurrent infections and clinical outcomes could not be established in our retrospective study, it clearly demonstrated the clinical significance of concurrent infections in patients with PVO. Our results, based on a large number of patients, can be practically used as a reasonable reference to warn clinicians of clinical risks due to concurrent infections in patients with PVO and help predict clinical outcomes.

## Figures and Tables

**Table 1:** Comparison of baseline patient characteristics.

Variables		Patients with Concurrent Infection	Patients without Concurrent Infection	*p*-Value
Number of patients		175	520	
Age		72.5 ± 9.7	68.1 ± 10.5	<0.001
Sex ratio (F:M)		89:86	279:241	0.521
BMI (kg/m^2^)		24.5 ± 3.8	25.1 ± 3.3	0.090
Follow-up days		1536 ± 1192	2384 ± 1094	
Charlson Comorbidity Index score		3.0 ± 2.6	2.1 ± 2.1	<0.001
	Cerebrovascular disease	37 (21)	97 (19)	0.470
	Coronary artery disease	46 (26)	103 (20)	0.071
	Chronic obstructive pulmonary disease	26 (15)	51 (10)	0.066
	Liver disease	48 (27)	84 (16)	0.001
	Mild liver disease	13 (7)	59 (11)	
	Moderate to severe liver disease	35 (20)	25 (5)	
	End-stage renal disease	28 (16)	54 (10)	0.046
	Diabetes	93 (53)	226 (43)	0.026
	Uncomplicated	54 (31)	191 (37)	
	Complicated	39 (22)	35 (7)	
Smoking	Nonsmoker	96 (55)	311 (60)	0.292
	Exit smoker	67 (38)	166 (32)	
	Current smoker	12 (7)	43 (8)	
White blood cell (×10^9^/L)	Initial	12457 ± 3772	11833 ± 4373	0.070
Erythrocyte sedimentation rate (ESR, mm/h)	Initial	68 ± 19	71 ± 18	0.105
C-reactive protein (CRP, mg/L)	Initial	72 ± 28	70 ± 22	0.275

PVO, pyogenic vertebral osteomyelitis; F, M; female, male; BMI, body mass index. The figures in brackets represent percentages.

**Table 2:** Comparison of infection profiles.

Variables		Patients with Concurrent Infection	Patients without Concurrent Infection	*p*-Value
Bacteremia/septicemia		113 (65)	252 (48)	<0.001
Causative organisms of PVO	*Staphylococcus aureus*	78 (45)	266 (51)	<0.001
	Methicillin resistant	11 (6)	124 (24)	
	Methicillin sensitive	67 (38)	142 (27)	
	Other gram-positive bacteria	32 (18)	161 (31)	
	Gram-negative bacteria	54 (31)	84 (16)	
	Others or mixed	11 (6)	9 (2)	
Primary region	Single	148 (85)	490 (94)	<0.001
	Mainly cervical	20 (11)	52 (10)	
	Mainly thoracic	54 (31)	164 (32)	
	Mainly lumbosacrum	74 (42)	274 (53)	
	Multiple	27 (15)	30 (6)	
Presence of abscess	Epidural abscess	129 (74)	379 (73)	0.830
	Abscess anterior to epidural space	92 (53)	263 (51)	0.648
	Abscess posterior to epidural space	83 (47)	306 (59)	0.008
Number of infected vertebral bodies	Within 2 vertebral bodies	129 (74)	408 (78)	0.195
	Over 3 vertebral bodies	46 (26)	112 (22)	
American Spinal Injury Association Scale grade	A	0 (0)	1 (0)	<0.001
	B	1 (1)	26 (5)	
	C	28 (16)	101 (19)	
	D	50 (29)	220 (42)	
	E	96 (55)	172 (33)	
Severity of infection by Pola et al.	Type A	14 (8)	23 (4)	0.114
	Type B	32 (18)	118 (23)	
	Type C	129 (74)	379 (73)	

PVO, pyogenic vertebral osteomyelitis. The figures in brackets represent the percentages.

**Table 3:** Comparison of treatment profiles.

Variables		Patients with Concurrent Infection	Patients without Concurrent Infection	*p*-Value
Surgical treatment	Overall surgery	48 (27)	211 (41)	0.002
	Early instrumentation (within 6 weeks)	38 (22)	165 (32)	0.012
	Pedicle or lateral mass screw	35 (20)	158 (30)	
	Cage	11 (6)	21 (4)	
	Plate	6 (3)	11 (2)	
Complications associated to long-term use of antibiotics	Intravenous catheter-related infection	10 (6)	16 (3)	0.112
	Neutropenia	7 (4)	31 (6)	0.324
Surgery related complications	Overall reoperation	12 (7)	21(4)	0.129
	Implant failure	5 (3)	14 (3)	0.908
Duration of antibiotics (days)		45 ± 16	53 ± 17	<0.001
Hospital stay (days)		55 ± 20	62 ± 19	<0.001
Recurrence	Occurrence	24 (14)	72(14)	0.965
Mortality	90-day mortality	29 (17)	12 (2)	<0.001
	1-year mortality	40 (23)	32 (6)	<0.001

The figures in brackets represent the percentages.

**Table 4:** Effect of concurrent infection on the recurrence and mortality.

Groups	Outcome	Types of Concurrent Infection	Univariable			Multivariable		
			Odds Ratios	95% Confidence Interval	*p*-Value	Odds Ratios	95% Confidence Interval	*p*-Value
All groups	Recurrence		0.99	(0.60–1.63)	0.965	0.49	(0.26–0.94)	0.030
	Mortality							
	90-day mortality		8.41	(4.19–16.90)	<0.001	7.70	(2.82–21.03)	<0.001
	1-year mortality		4.52	(2.73–7.47)	<0.001	4.10	(1.96–8.59)	<0.001
Subgroups ^1^	Recurrence	Multiple concurrent infection	3.58	(1.72–7.46)	0.001	3.38	(1.31–8.72)	0.011
		Septic arthritis/osteomyelitis of the extremities	0.75	(0.26–2.15)	0.589	0.97	(0.29–3.20)	0.961
		Urinary tract infection	2.13	(1.10–4.15)	0.026	1.54	(0.71–3.33)	0.277
		Intra-abdominal infection	2.09	(0.91–4.77)	0.081	1.04	(0.40–2.67)	0.938
		Pneumonia	0.96	(0.39–2.33)	0.923	0.40	(0.14–1.11)	0.078
		Central nervous system infection	1.10	(0.32–3.84)	0.876	1.50	(0.37–6.06)	0.566
		Cardiac infection	1.18	(0.34–4.11)	0.800	0.74	(0.17–3.13)	0.680
	90-day Mortality	Multiple concurrent infection	13.34	(6.09–29.19)	<0.001	9.42	(3.28–27.06)	<0.001
		Septic arthritis/osteomyelitis of the extremities	2.70	(0.99–7.34)	0.052	3.90	(0.95–15.95)	0.059
		Urinary tract infection	1.31	(0.45–3.81)	0.625	0.84	(0.25–2.83)	0.777
		Intra-abdominal infection	4.97	(2.02–12.27)	<0.001	2.91	(0.94–8.97)	0.063
		Pneumonia	6.69	(3.09–14.47)	<0.001	2.06	(0.73–5.86)	0.174
		Central nervous system infection	16.44	(6.36–42.50)	<0.001	27.50	(6.65–113.65)	<0.001
		Cardiac infection	4.61	(1.46–14.57)	0.009	6.17	(1.38–27.50)	0.017
	1-year mortality	Multiple concurrent infection	6.02	(2.89–12.56)	<0.001	3.22	(1.20–8.62)	0.020
		Septic arthritis/osteomyelitis of the extremities	1.74	(0.70–4.32)	0.235	2.08	(0.57–7.54)	0.266
		Urinary tract infection	1.32	(5.73–3.04)	0.515	0.83	(0.29–2.36)	0.727
		Intra-abdominal infection	3.57	(1.59–8.01)	0.002	2.27	(0.78–6.66)	0.134
		Pneumonia	4.61	(2.32–9.16)	<0.001	1.60	(0.60–4.25)	0.346
		Central nervous system infection	12.30	(4.91–30.85)	<0.001	14.05	(4.14–47.70)	<0.001
		Cardiac infection	2.38	(0.77–7.39)	0.132	2.83	(0.71–11.30)	0.142

The following variables were adjusted in all multivariable analyses: age, Charlson comorbidity index score, liver disease, diabetes, end-stage renal disease, bacteremia or septicemia, causative organisms, ASIA grade, surgical treatment, and early spinal instrumentation. ^1^ In the subgroup analysis, multivariable adjustment was additionally performed for other types of concurrent infections that showed intergroup differences between patients with and without concurrent target infections. The additionally adjusted variables for subgroup analysis are presented in Supplementary Table S1.

**Table 5:** Types of concurrent infections according to the diagnostic timings and their variance inflation factors.

Types of Concurrent Infections	Number of Patients	Before the PVO Diagnosis	After the PVO Diagnosis	Variance Inflation Factor
Septic arthritis/osteomyelitis of the extremities	37	16 (43)	21 (57)	1.16
Urinary tract infection	54	26 (48)	28 (52)	1.40
Intra-abdominal infection	33	20 (61)	13 (39)	1.27
Pneumonia	45	28 (62)	17 (38)	1.14
Central nervous system infection	20	12 (60)	8 (40)	1.15
Cardiac infection	19	14 (74)	5 (26)	1.22
etc.	14	11 (79)	3 (21)	1.18
Multiple concurrent infection	35	-	-	2.31
Overall single concurrent infection	140	80 (57)	60 (43)	-

The figures in brackets represent the percentages.

## Data Availability

The datasets generated for the current study are not publicly available, but the analyzing results are available from the corresponding authors on reasonable request.

## Supplementary Materials

The following supporting information can be downloaded at: https://www.mdpi.com/article/10.3390/jpm12040541/s1, Table S1: Identified causative organisms of pyogenic vertebral osteomyelitis in the whole cohort.
