# Supplementary material for: Clinical Characteristics of Patients with Pyogenic Vertebral Osteomyelitis and Concurrent Infections and Their Clinical Outcomes
Source: J Pers Med. 2022 Mar 29;12(4):541. doi: 10.3390/jpm12040541 (PMC9028400; doi:10.3390/jpm12040541)
Supplement: Supplementary file 1 [file jpm-12-00541-s001.zip › jpm-1658385-supplementary.pdf]

Clinical Characteristics of Patients with Pyogenic Vertebral Osteomyelitis and Concurrent Infections and Their Clinical Outcomes

Kim Donghyun, MD[a], Jihye Kim, MD, PhD[b], Tae-Hwan Kim, MD, PhD[a]

[a]Spine Center, Department of Orthopedics, Hallym University Sacred Heart Hospital, Hallym University College of Medicine, Anyang, Korea.

Postal address: 22 Gwanpyeong-ro, 170beon-gil, Dongan-gu, Anyang-si, Gyeonggi-do 14068, Republic of Korea.

[b]Division of Infection, Department of Pediatrics, Kangdong Sacred Heart Hospital, Hallym University College of Medicine, Seoul, Korea.

Postal address: 150, Seongan-ro, Gangdong-gu, Seoul, 05355, Republic of Korea.

Supplementary Table S1. Identified causative organisms of pyogenic vertebral osteomyelitis in the whole cohort

Types of bacteria	Category	Patients with concurrent infection	Patients without concurrent infection
Staphylococcus aureus	methicillin-resistant Staphylococcus aureus	11	124
	methicillin-sensitive Staphylococcus aureus	67	142
Other gram-positive bacteria	Coagulase-negative staphylococci	2	91
	Streptococcus pneumoniae	14	18
	Streptococcus agalactiae	3	24
	Streptococcus viridans	0	16
	Enterococcus faecalis	5	7
	Enterococcus faecium	8	5
Gram-negative bacteria	Escherichia coli	18	46
	Pseudomonas aeruginosa	6	25
	Acinetobacter baumannii	8	2
	Klebsiella pneumoniae	11	4
	Salmonella enterica	5	4
	Enterobacter cloacae	3	3
	Enterobacter aerogenes	2	0
	Other Enterobacteriaceae	1	0
Others or mixed		11	9
